# Differential transcriptomic changes in the central nervous system and urinary bladders of mice infected with a coronavirus

**DOI:** 10.1371/journal.pone.0278918

**Published:** 2022-12-09

**Authors:** Taylor C. Clarkson, Nao Iguchi, Alison Xiaoqiao Xie, Anna P. Malykhina

**Affiliations:** Division of Urology, Department of Surgery, University of Colorado Anschutz Medical Campus, Aurora, Colorado, United States of America; Universitat de Valencia Facultat de Medicina i Odontologia, SPAIN

## Abstract

Multiple sclerosis (MS) often leads to the development of neurogenic lower urinary tract symptoms (LUTS). We previously characterized neurogenic bladder dysfunction in a mouse model of MS induced by a coronavirus, mouse hepatitis virus (MHV). The aim of the study was to identify genes and pathways linking neuroinflammation in the central nervous system with urinary bladder (UB) dysfunction to enhance our understanding of the mechanisms underlying LUTS in demyelinating diseases. Adult C57BL/6 male mice (N = 12) received either an intracranial injection of MHV (coronavirus-induced encephalomyelitis, CIE group), or sterile saline (control group). Spinal cord (SC) and urinary bladders (UB) were collected from CIE mice at 1 wk and 4 wks, followed by RNA isolation and NanoString nCounter Neuroinflammation assay. Transcriptome analysis of SC identified a significantly changed expression of >150 genes in CIE mice known to regulate astrocyte, microglia and oligodendrocyte functions, neuroinflammation and immune responses. Two genes were significantly upregulated (*Ttr* and *Ms4a4a)*, and two were downregulated (*Asb2* and *Myct1*) only in the UB of CIE mice. *Siglec1* and *Zbp1* were the only genes significantly upregulated in both tissues, suggesting a common transcriptomic link between neuroinflammation in the CNS and neurogenic changes in the UB of CIE mice.

## Introduction

Multiple sclerosis (MS) is a chronic autoimmune disease characterized by the development of demyelinated lesions in the brain and spinal cord (SC), and progressive neurodegeneration that significantly changes patients’ personal, professional and social quality of life [1, 2]. Up to 90% of patients with MS develop neurogenic lower urinary tract symptoms (LUTS) such as detrusor overactivity, urgency, increased frequency of micturition, incontinence, incomplete emptying, hesitancy, weak urine stream, urinary retention, nocturia and dysuria [3, 4]. In 5–10% of MS patients, LUTS are present at the onset of the disease [3], and the severity of symptoms increases along with physical disability as the disease progresses [5]. Patients with chronic development of MS may experience severe LUTS that are often accompanied by bowel and sexual dysfunction [2, 6].

Several animal models of MS are available to study the pathophysiological mechanisms of the disease. Experimental autoimmune encephalomyelitis (EAE) is one of the well-established rodent models of MS, in which demyelination in the central nervous system (CNS) is induced by the treatment with antibodies against myelin binding protein (MBP), proteolipid protein (PLP), or myelin oligodendrocyte glycoprotein (MOG) [7–10]. In this model, animals develop pathological features of inflammation and demyelination in the CNS with increased immune cell infiltration and pro-inflammatory cytokine production [8–10]. Studies evaluating voiding function in EAE model detected symptoms of detrusor areflexia prior to EAE onset followed by detrusor overactivity at the later stages of the disease. In addition, the observed neurogenic LUTS is correlated with spinal cord inflammation and hindlimb paralysis [8, 11, 12]. The EAE model is considered to represent a "monophasic disease" characterized by limited neurodegeneration, chronic inflammation, and an unclear onset [13]. Demyelination in EAE is mediated mainly by CD4^+^ T cells [14], while CD8^+^ T cell driven immune mechanisms are more characteristic of human MS [15]. The described factors make it difficult to define a relationship between the level of neurodegeneration, disease onset and the severity of neurogenic LUTS, as well as complicate the ability to extrapolate the data from EAE model to MS patients [16, 17].

Human MS is a multifactorial disease that can be triggered by environmental factors, such as viral infections [18, 19]. A coronavirus-induced encephalomyelitis (CIE) in mice [20–22] is a validated animal model of MS induced by a single inoculation with a neurotropic A59 strain of mouse hepatitis virus (MHV) [23, 24]. The model reflects a spontaneous onset of neuroinflammation in the CNS with a robust innate immune response followed by adaptive immunity, involving CD8^+^ T-cells, CD4^+^ T-cells, and antiviral antibodies [23, 25, 26]. The long-term follow-up of CIE mice revealed three distinct phenotypes of neurodegenerative symptoms that closely resemble respective MS phenotypes in humans [27], supporting the translational relevance of this murine model. Our group previously characterized neurogenic LUTS in CIE mice, and identified several neural mechanisms contributing to the development of neurogenic bladder dysfunction [24, 27, 28]. The determined mechanisms included neurodegenerative changes in the CNS, activation of gliosis at the lesion sites, increased pro-inflammatory cytokine expression, and altered nerve-mediated contractions of the detrusor [24, 27, 28].

A better understanding of the cellular pathways triggering voiding dysfunction in MS, as well as identification of bladder specific molecular targets would provide a required knowledge foundation for the development of new therapies to alleviate LUTS in patients with neurodegenerative diseases. In this study, we aimed to identify key genes and pathways linking coronavirus-induced neuroinflammation in the CNS with UB dysfunction to clarify the mechanisms of neurogenic LUTS development in neurodegenerative disorders.

## Results

### Coronavirus-induced neuroinflammation triggers significant changes in gene expression profiles

**Fig 1:**
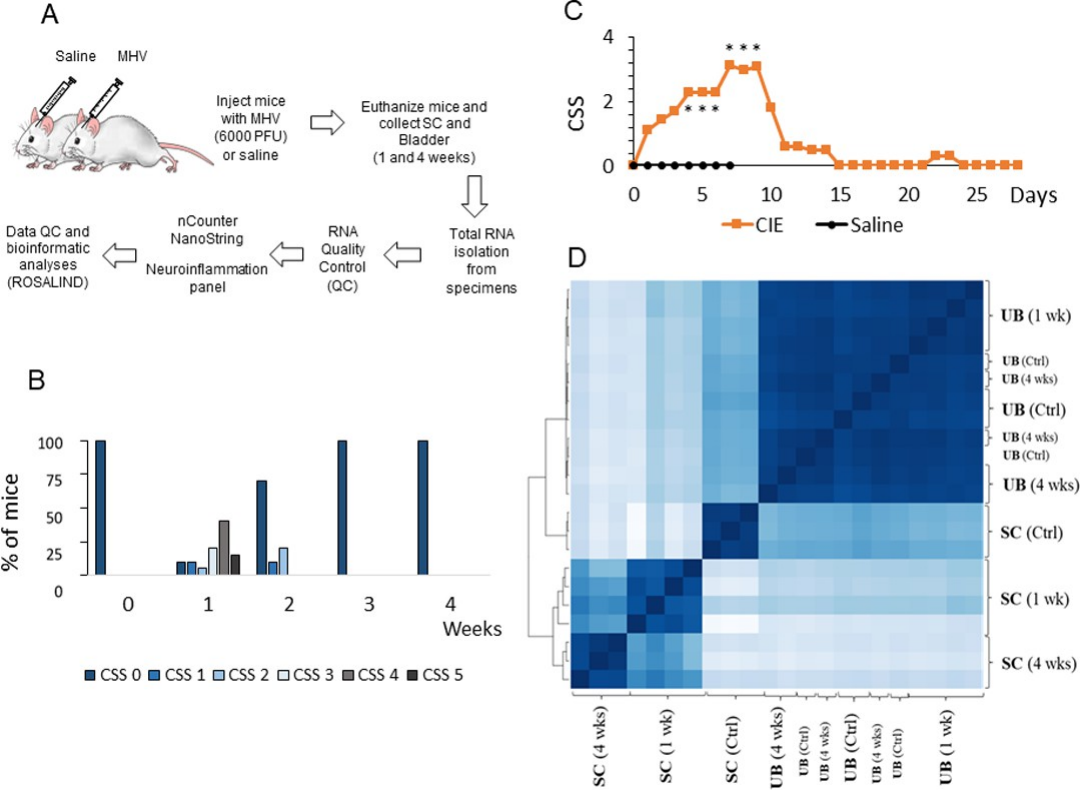
Experimental design of the study and clinical symptom scoring of the disease progression. A, Schematic presentation of experimental groups, time points and RNA analysis approach. B, Percentage of mice developed different levels of neurodegeneration, as evaluated by the clinical symptom scores (CSS) during experimental timeline. C, Average daily CSS values for CIE mice. D, Heat map of the genes expressed in the SC and UB samples isolated from control and CIE mice at 1 and 4 wks post-infection.

To evaluate the effects of coronavirus-induced neuroinflammation on gene expression profiles in the SC and UB samples, all mice were divided into 3 groups: control group (N = 4), CIE 1 wk group (N = 4) and CIE 4 wks group (N = 4). The workflow of experimental study design is included in Fig 1A. Analysis of CSS confirmed that CIE mice developed the most significant neurological impairment around 1 wk post-infection (Fig 1B), consistent with data previously published by our group [24, 27]. Approximately 85% of mice in the CIE groups developed a CSS of 3 or higher (Fig 1C, p ≤0.001 to control group). None of the control mice developed any clinical symptoms during the same period. In addition, CIE mice experienced a significant weight loss, peaking at 8 days post-infection with an average loss of 24% of their baseline body weight, while the control mice gained, on average, 0.5% of their baseline body weight at the same time point.

Lumbosacral (L6-S2) segments of the SC and UB were collected at 1 wk and 4 wks after MHV injection, followed by total RNA isolation from each specimen and subsequent run of the nCounter Neuroinflammation panel (Fig 1A). Two SC samples (one from control group and one from CIE 4 wks group) were excluded from further analyses due to quality flags detected by the software, indicating a low percentage of housekeeping genes in those samples.

A sample correlation heatmap was generated as part of the QC step (Fig 1D), reflecting the correlation values between the gene expression of the samples—with the darkest blue being the strongest correlation, and the lightest blue being the weakest. The heatmap analysis confirmed that genes in the UB samples clustered together regardless of the experimental group or control. The genes in UB samples from CIE 4 wks group clustered most closely with the control group, whereas the genes from CIE 1 wk group were most strongly correlated with each other. All SC samples strongly clustered together within each experimental group, indicating distinct differences in SC gene expression between the CIE mice and controls at both time points (Fig 1D).

### Time-dependent profile of neuroinflammatory genes in the SC of CIE mice

**Fig 2:**
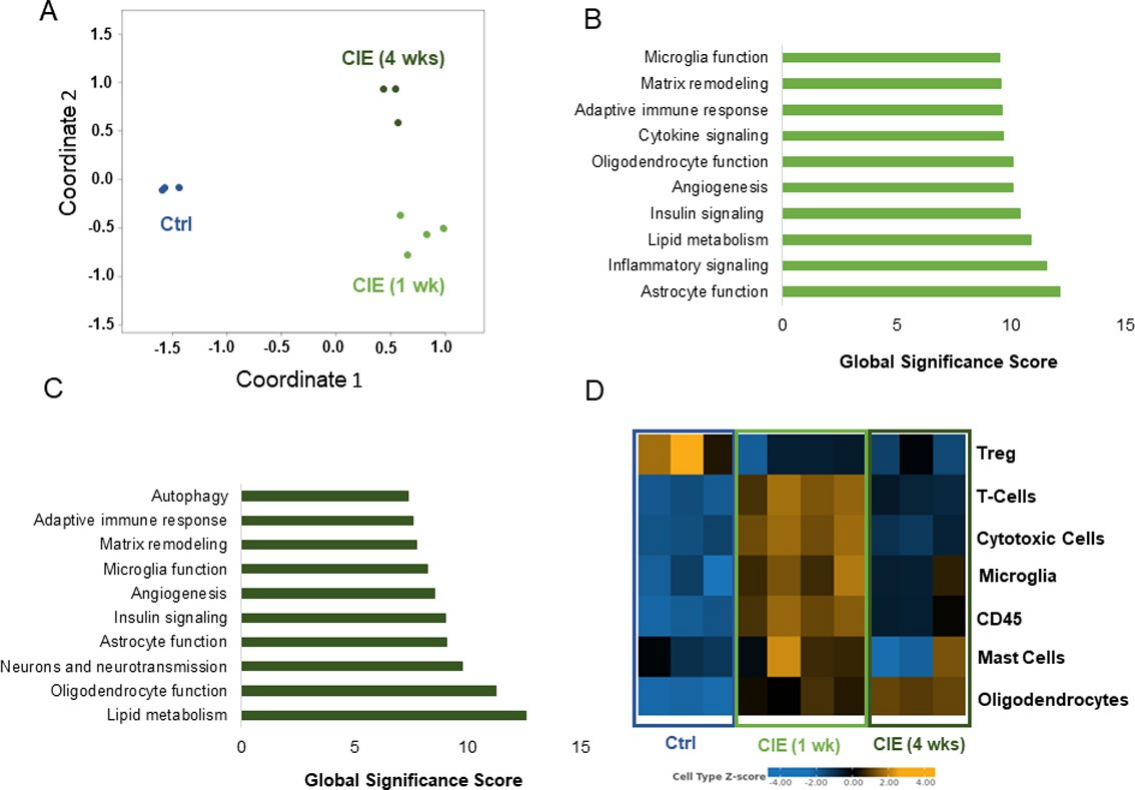
Cell type- and signaling pathway-specific changes in gene expression of SC specimens from control and CIE mice. A, A multi-dimensional scaling plot of the SC samples shows group-specific clustering of the genes in the control (Ctrl) group, and in both experimental groups (CIE 1 wk, and CIE 4 wks). The coordinates on the axis are arbitrary. This plot is presented in a way of displaying similarities and differences between data points. Samples that are clustered together generally have similar patterns of expression. Gene changes in top 10 signaling pathways in the SC cord of CIE mice at 1 wk (B) and 4 wks (C) post-infection. D, Heat map of the genes clustered based on their expression levels in different cell types (SC specimens).

In addition to the sample correlation heat map (Fig 1D), we also checked the clustering of the genes using a MDS plot (Fig 2A). As seen on the plot, the genes in the control, CIE 1 wk and CIE 4 wks groups clustered apart from each other, indicating distinct differences in the gene expression profiles among all groups. Next, we performed a gene set analysis (GSA) that summarized the global significance score (GSS) of the gene sets within a selected signaling pathway. Fig 2B and 2C present the top 10 GSSs for CIE 1 wk and CIE 4 wks groups, respectively. Since the total number of genes significantly up- or downregulated after infection with the virus was 440 at 1 wk and 406 at 4 wks, we used the cut off value of a +/-fold change of 10, and a p value of ≤ 0.05. Using this approach, we identified 179 genes at 1 wk and 154 genes at 4 wks significantly changed in the SC samples from CIE mice (S1 Table). Both CIE groups (1 wk and 4 wks) showed substantial changes in gene expression of the signaling pathways related to astrocyte function (GSS for CIE 1 wk = 12.2; CIE 4 wks = 9.1); matrix remodeling (GSS for CIE 1 wk = 9.6; CIE 4 wks = 7.8); adaptive immune response (GSS for CIE 1 wk = 9.6; CIE 4 wks = 7.6); oligodendrocyte function (GSS for CIE 1 wk = 10.1; CIE 4 wks = 11.3); angiogenesis (GSS for CIE 1 wk = 10.1; CIE 4 wks = 8.5); insulin signaling (GSS for CIE 1 wk = 10.4; CIE 4 wks = 9.0); and lipid metabolism (GSS for CIE 1 wk = 10.9; CIE 4 wks = 12.6) when compared to the control group. In addition, the changes in gene expression involved in cytokine (GSS = 9.7) and inflammatory (GSS = 11.6) signaling pathways were unique to the CIE 1 wk group, whereas the changes in neuron and neurotransmission genes (GSS = 9.8) as well as autophagy-related genes (GSS = 7.4) were unique for the CIE 4 wks group (S2 Table). These results suggest that CIE-induced neuroinflammation in the SC triggers long-lasting changes in transcriptional regulation of the genes involved in major MS-related signaling pathways (oligodendrocytes, microglia, astrocytes, adaptive immunity, angiogenesis, matrix remodeling and lipid metabolism) with reduction in cytokine and inflammatory pathways genes by 4 wks post-infection, and the recruitment of neuronal/neurotransmission signaling and autophagy pathways at the onset of demyelination in the CNS (4 wks).

The changes in gene expression profiles were most prominent in seven cell types present in SC samples: oligodendrocytes, CD45^+^ cells, mast cells, microglia, cytotoxic cells, T cells, and regulatory T cells (Treg). The heat map of gene expression changes specific for these cell types is based on Z-score (color labeling of up- or downregulation in gene expression, Fig 2D). Expression of multiple genes was mostly changed in oligodendrocytes (z = 0.62 at CIE 1 wk; z = 1.3 at CIE 4 wks; and z = -2.1 for control), CD45^+^ cells (z = 1.8 at 1 wk; z = -0.2 at 4 wks; and z = -2.2 for control), mast cells (z = 0.5 at 1 wk; z = -0.4 at 4 wks; and z = -0.3 for control), T cells (z = 1.6 at 1 wk; z = -0.5 at 4 wks; and z = -1.7 for control), Treg cells (z = -0.2 at 1 wk; z = -0.24 at 4 wks; and z = 0.5 for control), microglia (z = 0.8 at 1 wk; z = -0.02 at 4 wks; and z = -1.04 for control), and cytotoxic cells (z = 2.1 at 1 wk; z = -1.0 at 4 wks; and z = -1.8 for control). All abovementioned z scores were statistically significant between the experimental and control groups with p≤0.05.

**Fig 3:**
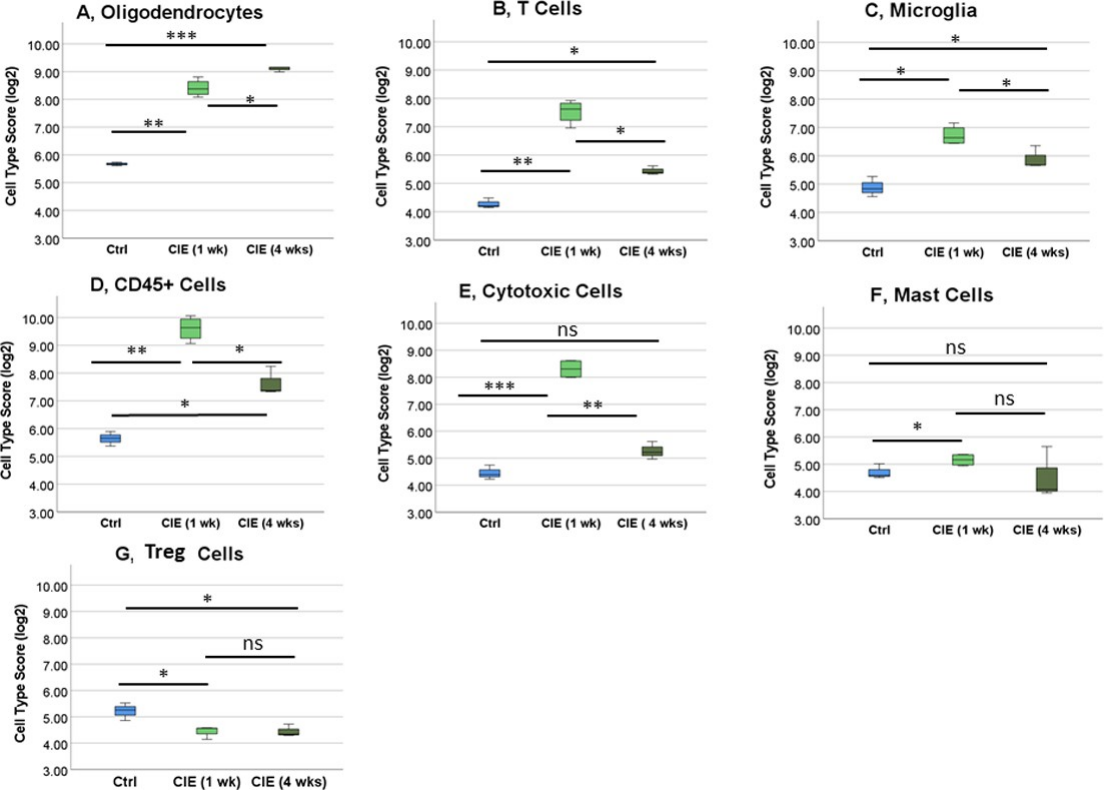
Clustering of the SC genes based on their expression level in different cell types. Cell type scores were significantly different between the control and experimental groups for several populations of the cells including oligodendrocytes (A), T cells (B), microglia (C), CD45+ cells (D), cytotoxic cells (E), mast cells (F), and Treg cells (G). n = 4 for CIE 1 wk group, n = 3 for CIE 4 wks and control groups. *p ≤0.05, **p≤0.01, ***p ≤0.001.

The cell type score was used to compare gene expression in specific populations of SC cells from different groups. In oligodendrocytes (Fig 3A), there was an initial 567% increase in gene expression levels in CIE 1 wk group (p<0.001 to control), followed by an additional 61% increase above 1 wk level in CIE 4 wks group (p<0.0003 to 1 wk). Expression of the genes in T cells (Fig 3B), microglia (Fig 3C), and CD45^+^ population (Fig 3D) was significantly increased in CIE 1 wk group followed by a decrease in expression levels at 4 wks. However, the cell type scores for T cells, CD45^+^ cells and microglia were still significantly elevated at 4 wks post-inoculation in comparison to the control group. Expression of the genes in cytotoxic cell population (Fig 3E) and mast cells (Fig 3F) was significantly up-regulated at 1 wk post-infection with the virus (by 1,343% in cytotoxic cells, p<0.0004; and by 36% in mast cells, p<0.04), followed by a decrease to control values at 4 wks. Treg cells (Fig 3G) experienced a significant downregulation in gene expression profiles in both CIE groups (by 40%, p<0.04). These data confirm substantial changes in immune and glial cells transcriptomes during acute stage of the infection, with many changes lasting for at least 4 wks. In comparison, cytotoxic and mast cells experienced transient changes in gene expression levels followed by the recovery to the baseline, whereas the gene profiles of Treg cells became significantly downregulated for the entire period of experimental observation.

### Coronaviral infection induced differential changes in UB genes in comparison to SC samples

**Fig 4:**
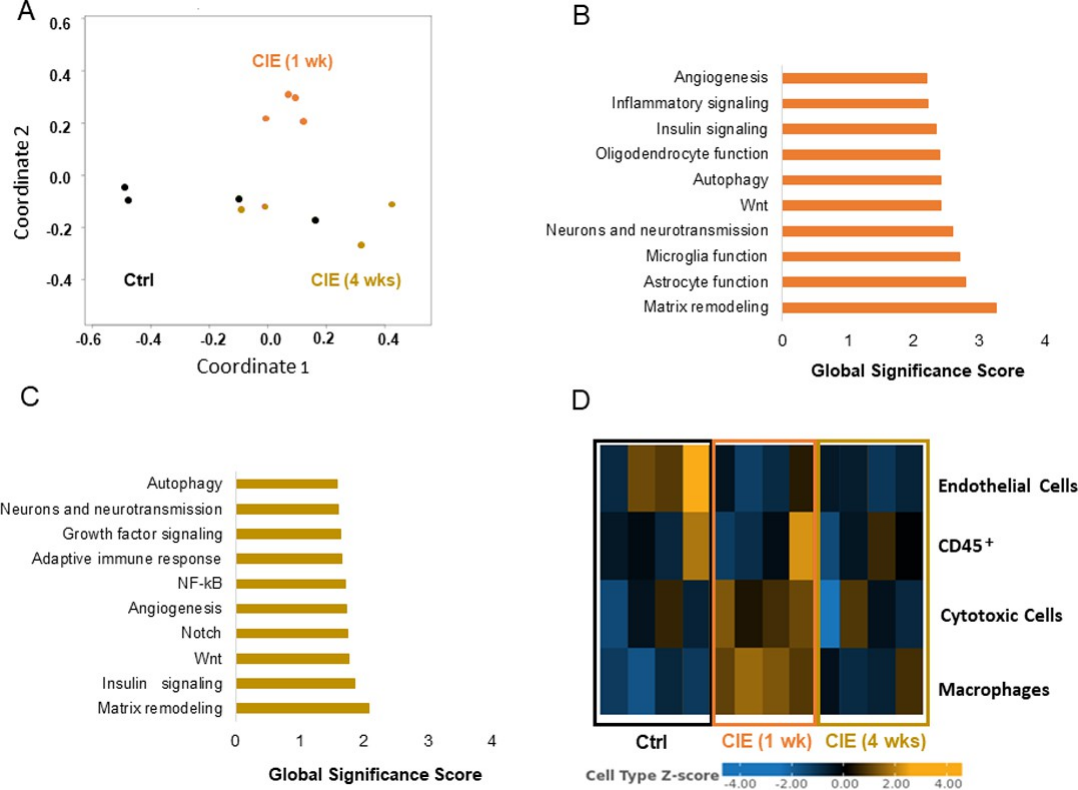
Cell type- and pathway-specific changes in the expression of the genes in the UB of control and CIE mice. A, A multi-dimensional scaling plot of the UB samples shows a different profile of gene expression in the CIE group at 1 wk post-infection, but less significant changes in gene expression between the control and CIE 4 wks group post-infection with a coronavirus. The coordinates on the axis are arbitrary. This plot is presented in a way of displaying similarities and differences between data points. Samples that are clustered together generally have similar patterns of expression. Global significance score (GSS) -based gene changes in the UB signaling pathways in CIE mice at 1 wk (B) and 4 wks (C) post-infection. D, Heat map of the UB genes clustered based on their expression levels in different cell types.

The same analysis of the individual genes, cell profiles and signaling pathways was performed in UB samples. In the UB, the statistically significant changes were detected in a smaller population of the genes, therefore, we filtered UB genes using a +/- fold change of 1.5 and a p value of ≤0.05, leading to the detected changes in 45 out of 770 genes in the UB of CIE 1 wk group, and 31 genes in the CIE 4 wks group. The analysis of MDS plot (Fig 4A) revealed similarities in gene expression between control and CIE 4 wks group with some points located very close to each other. However, expression of the genes in CIE 1 wk group was clustered together, and away from the other two groups. This data suggests that while there is a significant change in UB gene expression at 1 wk post-infection with MHV, there is a trend towards recovery to control values by 4 wks after the infection.

Analysis of GSA in UB samples showed differential changes in gene expression based on the GSS of specific pathways (Fig 4B and 4C). In the bladder, the signaling pathways most affected by the MHV infection at both time points (1 and 4 wks) included matrix remodeling (GSS for CIE 1 wk = 3.2; CIE 4 wks = 2.1); neurons/ neurotransmission (GSS for CIE 1 wk = 2.6; CIE 4 wks = 1.6); *Wnt* signaling (GSS for CIE 1 wk = 2.4; CIE 4 wks = 1.8); autophagy (GSS for CIE 1 wk = 2.4; CIE 4 wks = 1.6); angiogenesis (GSS for CIE 1 wk = 2.2; CIE 4 wks = 1.7); and insulin signaling (GSS for CIE 1 wk = 2.3; CIE 4 wks = 1.9). Expression of the genes involved in astrocyte function (GSS = 2.8), microglia function (GSS = 2.7), oligodendrocyte function (GSS = 2.4), and inflammatory signaling (GSS = 2.2) was significantly changed only at 1 wk post-infection with the virus (Fig 4B). In the CIE 4 wks group, the changed pathways included *Notch* signaling (GSS = 1.7), *NF-kB* signaling (GSS = 1.7), adaptive immune response (GSS = 1.7), and growth factor signaling (GSS = 1.6, Fig 4C). These GSS indicated changes in UB gene expression and pathway activation between the two CIE groups when compared to each other, and to the control group.

The directed significance score (DSS) of several signaling pathways was used to determine whether involved genes were primarily up- or downregulated at each time point. Expression of the genes in matrix remodeling pathway (DSS for CIE 1 wk = -1.9; CIE 4 wks = -0.8) and angiogenesis (DSS for CIE 1 wk = -1.6; CIE 4 wks = -0.6) was primarily downregulated at both time points. Expression of the UB genes was mostly upregulated at both time points in insulin signaling pathway (DSS for CIE 1 wk = 1.1; CIE 4 wks = 0.5) and neurons/neurotransmission (DSS for CIE 1 wk = 1.3; CIE 4 wks = 0.7). In addition, gene expression of autophagy pathway (DSS for CIE 1 wk = -1.2; CIE 4 wks = 0.5) and *Wnt* signaling (DSS for CIE 1 wk = -2.4; CIE 4 wks = 0.8) was down-regulated at 1wk post-infection followed by up-regulation at 4 wks time point. The gene expression changes detected exclusively in 1 wk CIE group were all downregulated and included astrocyte function (DSS = -1.5); microglia function (DSS = -0.9); oligodendrocyte function (DSS = -1.2); and inflammatory signaling (DSS = -0.54; S3 Table). Expression of the genes in pathways changed solely at 4 wks post-inoculation included *Notch* signaling (DSS = 0.8); *NF-kB* signaling (DSS = 0.9); adaptive immune response (DSS = -0.9); and growth factor pathway(DSS = -0.3; S4 Table).

**Fig 5:**
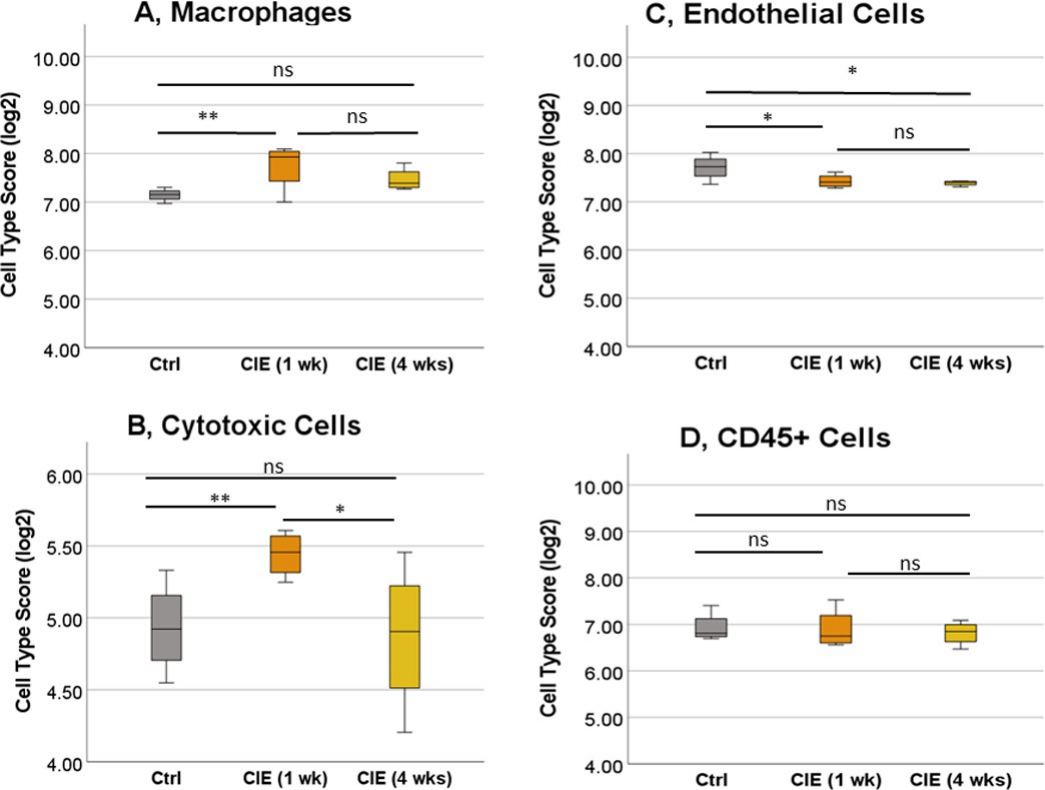
Differential expression of the genes in cell types present in the bladder wall. Cell type scores were significantly different between the control and experimental groups for several populations of the cells including macrophages (A), cytotoxic cells (B) and endothelial cells (C). No significant change in gene expression between the groups was noted for CD45^+^ cells (D). N = 4 for each group. *p ≤0.05, **p≤0.01.

The cell type analysis of UB samples established that, unlike in the SC, expression of the genes was changed in only four major cell types (Fig 4D). Gene expression in three cell populations—macrophages, cytotoxic cells and endothelial cells—was significantly changed in the UB in CIE 1 wk group in comparison to the control (Fig 5). There was a variability in expression profiles in CD45^+^ cells between the groups, however, those changes did not reach the level of statistical significance. Among significantly changed cell types, acute infection with the virus (1 wk) led to an upregulation of the genes in macrophages (by 76% to control, p<0.001, Fig 5A) and cytotoxic cells (by 42% to control, p<0.018, Fig 5B) followed by the recovery to control values at 4 wks post-infection. However, the gene expression profiles were significantly downregulated in endothelial cells in both CIE 1 wk (18% decrease to control, p<0.03) and CIE 4 wks groups (Fig 5D).

### Expression of the genes changed only in the UB, and similarities in transcriptomic profiles between the two organs

**Fig 6:**
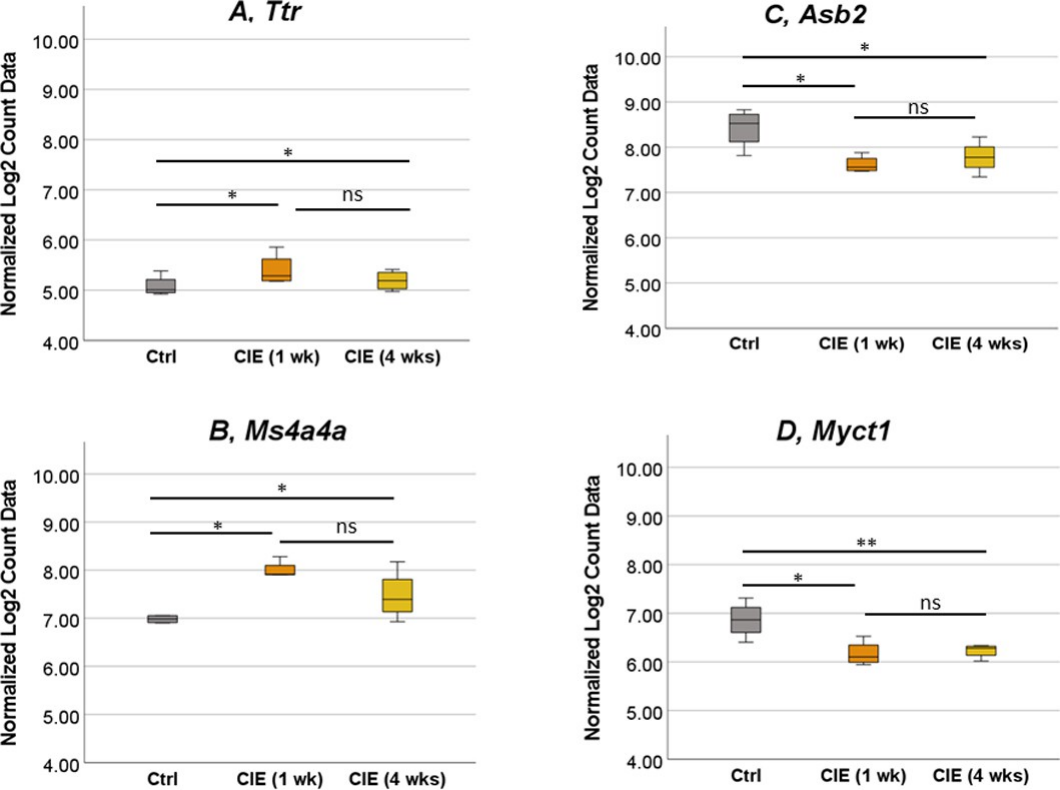
Expression of the genes changed only in the UB of CIE mice. Two genes, *Ttr* (A) and *Ms4a4a* (B) were upregulated only in UB samples of CIE 1 wk mice. Additional two genes, *Asb2* (C) and *Myct1* (D) were downregulated only in UB samples from CIE 1 wk group. The same significant changes were observed for all respective genes in UB samples from CIE 4 wks groups. N = 4 for each group. *p ≤0.05, **p≤0.01.

Next, we performed a detailed analysis of UB samples to select genes significantly changed only in the UB, but not in the SC of CIE mice. We identified significant differences in the expression of four UB genes. These genes included *Ttr* (Fig 6A), *Ms4a4a* (Fig 6B), *Asb2* (Fig 6C), and *Myct1* (Fig 6D). *Ttr* gene was upregulated by 2.2-fold in CIE 1 wk group (p<0.036, Fig 6A), and by 1.7-fold at 4 wks in the UB samples. *Ms4a4a* had a 2.3-fold increase at 1 wk (p<0.015, Fig 6B), and 1.6-fold change at 4 wks post-infection (p<0.011, Fig 6B). In contrast, *Asb2* and *Myct1* genes were both downregulated in the UB samples at both, 1 wk and 4 wks time points after infection. *Asb2* had a -1.8 fold change in CIE 1 wk group (p<0.036, Fig 6C), and a -1.6-fold change in the CIE 4 wks group (p<0.013, Fig 6C). *Myct1* gene had a -1.7- fold change in the CIE 1 wk group (p<0.039, Fig 6D), and a -1.6-fold change in the CIE 4 wks group (p<0.0055, Fig 6D).

**Fig 7:**
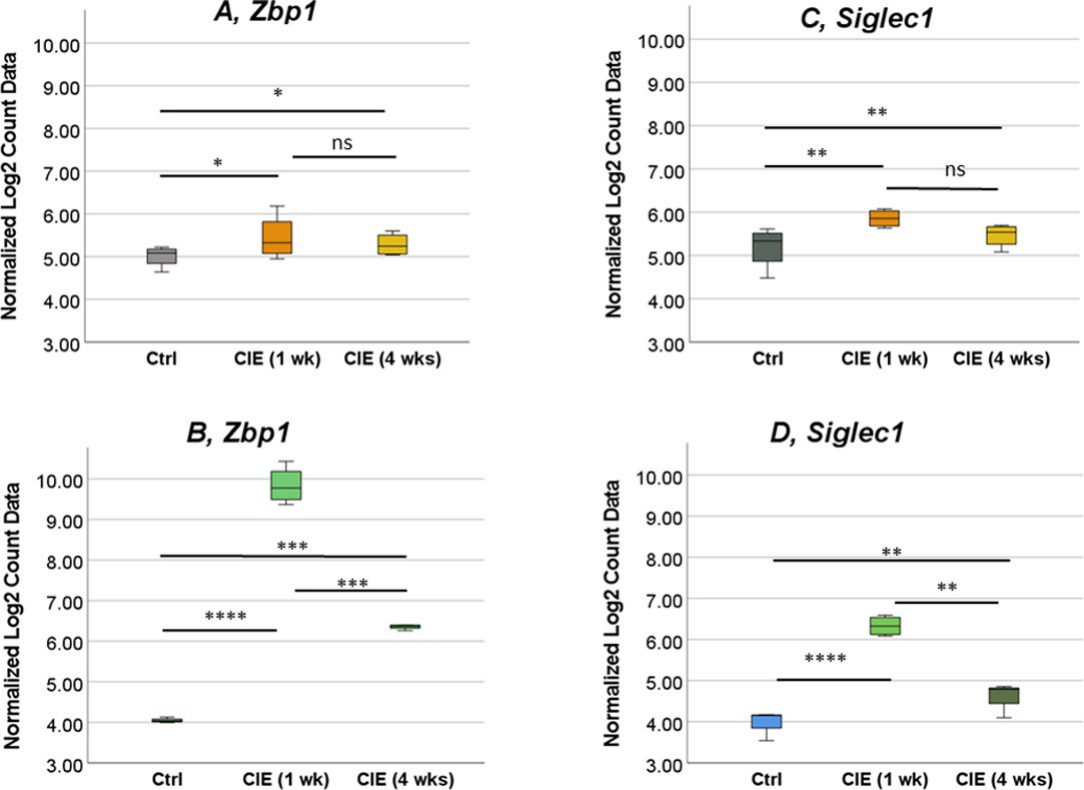
Up-regulation of *Zbp1* and *Siglec1* genes in both, SC and UB, samples from CIE mice. The two genes, *Zbp1* (A and B) and *Siglec1* (C and D), were upregulated in both, SC (B and D) and UB (A and C), specimens in CIE mice. *p ≤0.05, **p≤0.01, ***p ≤0.001.

Only two genes significantly changed expression levels in both tissues at both time points–*Zbp1* (Fig 7A and 7B), and *Siglec1* (Fig 7C and 7D). *Zbp1* and *Siglec1* genes had significantly increased expression in both, the UB and SC samples at 1 wk and 4 wks time points in CIE mice. For both genes in both organs, the level of expression was higher in CIE 1 wk group, followed by a decrease at 4 wks. In the SC, *Zbp1* expression was increased by 942-fold at 1 wk and 69-fold at 4 wks (Fig 7A). Expression of *Zbp1* gene in the UB was increased by 2.8-fold and 2.2-fold at 1 wk and 4 wks post-infection, respectively (Fig 7B). In the SC, *Siglec1* expression was increased by 71-fold at 1 wk, and 12-fold at 4 wks post-infection (Fig 7C). The level of *Siglec1* gene expression in the UB was a 2.6-fold increase at 1 wk, and 1.8-fold at 4 wks after the infection.

## Discussion

Neuroinflammation and neurodegeneration in the CNS are the main hallmarks of human MS. Viral models of neuroinflammation were previously used for studying MS pathogenesis [18, 29], and several strains of mouse hepatitis virus (MHV, *Coronaviridae* family) were proven to trigger severe neurodegeneration in the CNS similar to human MS [22, 30]. Prior studies from our group confirmed that mice inoculated with MHV develop acute inflammation in the CNS followed by progressive demyelination in the SC [24, 28]. The CIE mice also present with a significant neurologic deficit associated with voiding dysfunction that is comparable with neurogenic LUTS observed in MS patients [27]. In the present study, we performed a comprehensive evaluation of gene expression profiles in the SC and UB tissues from CIE mice, and identified several cellular targets and signaling pathways that can influence plasticity of the neural pathways innervating the lower urinary tract.

Transcriptomic analysis of neuroinflammatory gene expression in the SC and UB of CIE mice revealed a tissue-specific recruitment of several cell types and associated signaling pathways during the neurologic symptom development. The main signalling pathways affected by the infection in the SC included several glial populations—oligodendrocytes, astrocytes and spinal microglia. Most of the genes associated with oligodendrocyte function were significantly up-regulated at both 1 wk and 4 wks time points, and included *Plp1*, *Mobp*, *Mag*, *Myrf* and *Bcas1* genes. The *Plp1* gene encodes a major componenet of myelin which plays key role in the development of oligodendrocytes and maintenance of myelin sheath integrity [31]. Autoimmunity against Plp1 has been detected in MS pathology [32], and an increase of Plp1 phagocytoses by macrophages in active MS lesions was suggested to be an indicatior of ongoing demyelination [33]. *Mobp* gene, coding for the third most abundant protein in myelin, helps stabilize the myelin sheath, and was suggested as another potential MS autoantigen [32, 34]. *Mag*, an oligodendrocyte specific marker gene, is a member of the immunoglobulin superfamily involved in myelination, and expressed by oligodendrocytes in remyelinating plaques [35]. Mag peptides are recognized by B and T cells in MS, and were shown to inhibit neuronal growth [36] and contribute to MS progression [37]. *Myrf* and *Bcas1* genes were found to be upregultaed in new oligodendrocytes during remyelination and reflect on the process of active remyelination or myelin repaire in the CNS [38–40]. We also detected 2 genes in the SC that were exclusively upregulated at 4 wks post-infection. One of them, *Opalin*, is critical for oligodendrocyte differentiation, and plays a role in remyelination [35]. It is also a marker for remyelinating oligiodendrocytes, along with *Mog* gene [41] found to be solely up-regulated in the CIE 4 wk group, and *Mag (*mentioned above). The upregulation of remyelination-associated genes at 1 wk and 4 wks post-infection with MHV suggests an early onset of activation of reparative remyelination mechanisms in the CNS, which could be linked to the improvements in CSS scoring in CIE mice observed after 1 wk post-infection.

Astrocytes are known to be involved in the active development of MS lesions, and allow peripherial immune cells to infiltrate the CNS [42]. Astrocytes express *S100b* gene [43] under normal and pathophysiological conditions with the respective protein serving as an established biomarker of ongoing neurodegeneration [44]. The concentration of S100B reaches nanomolar range under stress conditions such as acute neuroinflammation, upon which S100B has a neurotoxic effect, and behaves as a damage-associated molecular pattern molecule [45]. Elevated levels of S100B were detected in cerebrospinal fluid (CSF) [44] and sera [46] of MS patients in the acute phase of the disease, and also in active demyelinating and chronic active MS plaques [47]. In animal models of neurodegeneration, a marked astrocytic elevation of S100B was observed upon demyelination, while inhibition of S100B action reduced demyelination and downregulated the expression of inflammatory molecules [46]. Significantly upregulated *S100b* expression in SC of CIE mice observed in our study (821-fold at 1 wk and 1700-fold at 4 wks) confirmed severe ongoing neuroinflammation in the CNS associated with demyelination and neurodegeneration. *Cxcl10*, another neuroinflammatory gene that was significantly upregulated in the SC samples, is also expressed in astrocytes and perivascular lymphocytes, and encodes one of the α-chemokines [48, 49]. Most of α-chemokines attract neutrophils, whereas CXCL10 and CXCL9 attract T cells which play a critical part in the pathogenesis of MS. A significant increase in CXCL10 was detected in the CSF of patients with relapsing-remitting MS in comparison with secondary progressive MS [50–52]. Recently, it was determined that CXCL10 is the most up-regulated chemokine associated with cytokine storm in COVID-19 infected patients [53].

Inflammatory signaling, a critical component of MS associated neuroinflammation, was significantly changed in SC samples from CIE mice along with the populations of inflammatory cells. The extensive list of the chemokine genes upregulated in CIE mice indicates that MHV infection causes a massive cytokine storm, as was previously reported for HIV and COVID-19 infections [54, 55]. *Ccr5*, *Ccl2*, *Ccl5*, and *Ccl7* were among the most significantly upregulated chemokine genes in SC samples at 1 wk post-infection. Chemokines regulate migration of T cells and macrophages into the CNS followed by subsequent activation and effector functions. CCR5 is a chemokine receptor of CC motif that is expressed on circulating Th1 cells [56, 57] and perivascular cells of monocyte/macrophage/microglial lineage in MS lesions [58, 59]. CCR5 was shown to be upregulated in and around lesion sites in MS patients [60, 61]. CCR5 ligands were also overexpressed in MHV-induced demyelination [62], and antisera to CCR5 reduced infiltration of lymphocytes in MHV infection [63]. Additional chemokines—*Ccl5*, *Ccl7*, and *Ccl2*—are highly involved in the pathology of MS as they recruit inflammatory cells to active MS lesions [64]. They are often found in increased numbers in active lesions, expressed by resident glia and perivascular leukocytes in human MS [52, 64, 65]. *Ccl2* gene is expressed in T cells, macrophages, synovial fibroblasts, platelets, and tubular epithelium, and promotes the migration and trafficking of leukocytes, including monocytes, T cells and NK cells [66]. Ccl5 has a very similar function to Ccl2 by triggering recruitment of leukocytes into inflammatory sites and stimulating activation of specific NK cells to produce chemokine-activated killer cells [67]. *Ccl7* gene is expressed by stromal cells, airway smooth muscle cells, and keratinocytes, acting as chemoattractant for leukocytes, including monocytes, basophils, eosinophils, basophils, NK cells, DCs, and activated T lymphocytes [68].

Among other groups of upregulated genes in the SC of CIE mice were toll-like receptors (*Tlr 2* and *Tlr7*). Toll-like receptors (TRL) play a critical role in modulating cytokine and chemokine secretion in response to exogenous pathogens [69]. *Tlr2* is expressed in endothelial cells of the CNS, microglia, astrocytes, oligodendrocytes [3, 24] and infiltrating cells in human MS. It was found to be upregulated in CSF and demyelinating lesions of MS patients [8, 18, 70, 71], as well as in animal models of MS at various disease stages [72–74]. *Trl7* is expressed in monocytes and macrophages, plasmacytoid DC and B cells, and TRL7 binds to single-stranded (viral) RNA [75]. Some MS patients express elevated mRNA levels of TLR7 at the onset of the disease, suggesting an early involvement of this receptor in the pathogenesis of MS development [76].

Multiple genes associed with adaptive immune response displayed changed expression in the SC of CIE mice, with most of them being upregulated at 1 wk post-infection with the virus. This group of genes included *Cd8a Cd3g* and *Siglec1*. *Cd8a* ecodes for a protein that mediates cell-to-cell interactions within the immune system, and is expressed by cytotoxic T lymphocytes [77]. *Cd3g* gene encodes the protein which is part of the CD3 T-cell receptor, along with *Cd3e* and *Cd3d* (also found to be upregulated, but only in the 1 wk SC group), shown to be important for antigen recognition [78]. *Siglec1* has increased expression in CD14^+^ monocytes in MS patients, specifically those with the progressive disease form [79], and was shown to play a role of a promoter for neuroinflammation in EAE models of MS [80].

Different patterns of gene expression were observed in the UB of CIE mice in comparison to SC samples. Out of 45 significantly changed genes, 20 were upregulated and 25 were downregulated in the UB in CIE 1 wk group. Among the upregulated genes, the top 5 included *Gzma*, *Fkbp5*, *Cd163*, *Ccl5*, and *Zbp1*, with some of these genes having established links with MS pathogenesis. For instance, *Fkbp5* gene encodes an immunophilin protein expressed in neurons, ependymal cells, and astrocytes [81]. This protein was significantly upregulated in the peripheral blood of MS patients, and in the spleens of EAE mice [82]. Other genes were found to have associations with MS and bladder dysfunction. *Cd163* gene is exclusively expressed in monocytes and macrophages, promotes local inflammation, and is a well-known biomarker for MS [83]. Increased expression of CD163 was linked to bladder pain and presence of inflammation in the urinary bladder [84]. *Ccl5* gene, also found to be upregulated in the SC in CIE 1 wk group, was detected at increased levels in the urine of interstitial cystitis patients [85, 86] and was associated with voiding dysfunction in these patients. Human MS GWAS studies identified a significant correlation between elevated plasma levels of Granzyme A (GzmA), encoded by *Gzma* gene, and the presence of MS diagnosis [87]. GzmA is secreted by cytotoxic lymphocytes, and has traditionally been viewed as a mediator of cell death. However, a growing body of data suggests that the physiological role of GzmA is promotion of inflammation. Plasma levels of GzmA were significantly elevated in patients with dengue fever and cytomegalovirus infection, in chikungunya virus (CHIKV) patients, and in patients with respiratory syncytial virus infection [88–91]. Additional studies established that subcutaneous injection of enzymatically active recombinant mouse GzmA was able to promote inflammation, both locally, at the injection site, as well as at a distant site [92]. Increased extracellular GzmA was able to disrupt basement membrane proteins *in vitro*, and cleave the α2(IV) chain of collagen IV in mice [93]. Additionally, extracellular GzmA cleaves fibronectin, MBP and heparin sulfate proteoglycans *in vitro*, suggesting its role in extracellular matrix (ECM) remodeling [93–98]. Destruction of ECM integrity may, in turn, contribute to the migration of activated cytotoxic T lymphocytes through the affected tissue [93, 99], followed by increased shedding of the epithelial cells due cleavage of ECM proteins, and impairment of epithelial integrity in the urinary mucosa. The mentioned transcriptomic changes point towards initial signs of neurogenic inflammation development in the UB followed by ECM remodeling and impairment of epithelial integrity in the bladder wall.

The top 5 downregulated genes in the UB of CIE mice at 1 wk post-infection included *Igf1*, *Col6a3*, *Myrf*, *Itga7*, and *Serpinf1*. The IGF1, encoded by *Igf1* gene—the most downregulated gene in the UB 1 wk group, has a function similar to insulin, and mediates cellular growth and development. It can stimulate remyelination [100, 101], and had increased expression in the UB of mice with spinal cord injury, as it stimulates bladder wall remodeling [102]. Downregulation of *Igf1* gene along with *Col6a3*, (encodes protein that is part of type VI collagen), *Itga7* (expressed in smooth muscle cells and mediates interactions with the cell matrix [103]) and simultaneous abovementioned upregulation of *Gzma* in the UB of CIE mice suggest significant alterations in the matrix remodeling of the UB, and could lead to a reduced capability of the bladder wall to regenerate which, in turn, could be one of the factors contributing to the occurrence of neurogenic voiding dysfunction. Interestingly, an upregulated *Gzma* expression in the UB from CIE mice occurred in parallel with downregulation of *SerpinF1* gene. Prior studies established that several peptides from SERPIN family serve as intra- and extracellular inhibitors for GzmA, therefore, their limited expression due to downregulation of the respective genes could indirectly promote the function of cytotoxic cells expressing GzmA protein [104]. Disruption of matrix integrity in the UB of the CIE mice fits with another important observation of our study regarding several genes being downregulated in endothelial cells during both, acute and late phases of the infection (Fig 5C). Endothelial cells were the only group of cells in the UB showing a long-term decrease in cellular function, thereby, supporting progressive diminution of urothelial integrity and permeability in CIE mice, which are often observed in neurogenic bladders [105–107].

The long-term follow up of transcriptomic changes in the UB of CIE mice (4 wks post-infection) revealed a distinct transcriptomic profile. In this group, 20 genes were significantly upregulated and 11 were downregulated in the UB tissue. Only two genes were upregulated in the UB of both CIE groups (1 wk and 4 wk), but not in the SC tissues—*Ttr* and *Ms4a4a*. *Ttr* gene encodes protein transthyretin which is mainly produced by the liver, and participates in transporting thyroid hormone thyroxine from blood to CSF [108]. Thyroxine helps oligodendrocyte precursor cells to turn into myelinating oligodendrocytes, thereby, enhancing remyelination after initial insult. Previous studies established that elevated serum levels of transthyretin were associated with severity of disability in MS patients [109], and that increased thyroid hormone administration enhances remyelination [110]. Additionally, posttranslational oxidative modifications to transthyretin correlated with low levels of thyroxine in the CSF and disease duration in MS patients [111]. Limited data are available to link thyroxine effects with bladder function/dysfunction, with only one report showing that systemic thyroxine treatment enhanced the relaxation of UB strips *in vitro* after stimulation with isoproterenol, and that the response was correlated with a hormone-induced increase in beta-adrenergic receptors in the bladder tissue [112]. *Ms4a4a* gene belongs to a family of *Ms4a* genes which encode transmembrane proteins that are mainly expressed in microglia [113]. The functional role of MS4A4A was discussed in the pathogenesis of Alzheimer’s disease with no publications linking *Ms4a4a* gene with voiding dysfunction or MS pathology [114, 115].

*Asb2* and *Myct1* were the two genes that were downregulated only in the UB samples. *Asb2* is important for the adaptive immune response [116], and prior studies reported that the Asb2 ubiquitin ligase activity drives degradation of the filamins [117]. Asb2 has two isoforms, hematopoietic-type ASB2α and muscle-type ASB2β, exerting E3 ubiquitin ligase activity toward filamin A and filamin B, respectively [118]. Filamin is expressed in the rat bladder, and was increased in the hypertrophic bladders in the partial bladder outlet obstruction model [119]. The knockdown of *Asb2* gene results in embryonic lethality [120], and there are no studies linking this gene to MS pathology. The second gene downregulated exclusively in UB samples was *Myct1*. It is a direct transcriptional target gene of c-Myc, and *Myct1 per se* can regulate the expression of various c-Myc target genes, thereby, recapitulating many c-Myc functions in the cells [121, 122]. A recent study established that in vascular smooth muscle, MYCT1-dependent downregulation of ribosomal proteins compromised the protein translational capacity of the cells for collagen production [123]. In addition, the endogenously expressed MYCT1 in vascular smooth muscle cells was involved in maintaining normal cellular functions including survival, proliferation and migration [123]. Whether the same mechanism might be present in bladder smooth muscle cells is yet to be established.

We identified two genes, *Zbp1* and *Siglec1*, to be significantly upregulated in both tissues, the SC and the UB, in CIE mice at both time points. *Zbp1* gene encodes a Z-DNA binding protein, and plays a role in the innate immune response by binding to foreign (mostly viral) DNA and RNA [124–127]. The downsteam signalling includes type-I interferon production followed by inflammasome activation, proinflammatory responses, and cell death [128]. In addition, *ZBP1* was found to be highly expressed in patients with systemic lupus erythematosus, and significantly correlated with immune cell infiltration in patients`specimens suggesting its potential role on triggering autoinflammatory conditions [129]. *Siglec1* gene encodes a member of the immunoglobulin superfamily expressed mostly in monocytes and some macrophages. Elevated SIGLEC1 protein expression was found in the brain but not in the blood of MS patients [130]. However, expression of SIGLEC1 by CD14^+^ monocytes was significantly increased in MS patients with the progressive form of the disease [79]. SIGLEC1^+^ macrophages in EAE model infiltrate the CNS, bind Treg cells and negatively regulate their expansion, leading to MS-like symptoms [131]. SIGLEC1^+^ cells promote neuroinflammation, and SIGLEC1+ phagocytes play key role in MS pathophysiology as was established in EAE mouse model of MS [80]. Circulating monocytes and macrophages could be one of the reasons that *Siglec1* expression was found to be elevated not only in the CNS, but also in the UB in our study.

Overall, we identified several cellular genomic targets linking neurodegenerative changes in the CNS with the development of neurogenic LUTS. The revealed transcriptomic datasets suggest correlations between extensive neuroinflammatory reaction in the SC caused by the virus with altered gene expression in the UB wall. Further studies are warranted to address functional significance of the identified genes, confirm tissue findings at the cellular level followed by the analyses of morphological and spatial relationships between the cells and cellular proteins.

## Methods and materials

### Animals and experimental groups

The study used 12 adult male C57BL/6J mice (N = 12, 12 wks of age) purchased from Jackson Laboratory (Bar Harbor, ME). Mice were housed in a temperature-regulated animal facility at the University of Colorado, Anschutz Medical Campus (CU-AMC) vivarium on a 14-hour light/10-hour dark cycle. All mice had *ad libitum* access to food and water with special measures taken to accommodate neurologically compromised animals. Animal procedures were performed according to the protocols approved by the University of Colorado Institutional Animal Care and Use Committee in accordance with relevant guidelines and regulations (IACUC #00472). The reporting of experimental animal data in the manuscript follows the recommendations in the ARRIVE guidelines (PLoS Bio 8(6), e1000412,2010).

### Mouse model of coronavirus-induced encephalomyelitis (CIE)

The mice received either a single intracranial injection of 20 μl sterile saline (N = 4, control group) or mouse hepatitis virus (MHV, A59 strain, 6000 PFU, N = 8, CIE group) in 20 μl sterile saline under isoflurane anesthesia, as previously described [24, 132]. After viral injection, mice were weighted and monitored daily for assessment of neurological symptom development by using clinical symptoms score (CSS). Clinical symptom score was used to compare neurodegenerative symptom development based on the following scale: 0 = normal with no clinical signs, 1 = loss of tail tonicity/kyphosis, 2 = tail paralysis/severe kyphosis, 3 = partial hindlimb paralysis, 4 = complete hindlimb paralysis, and 5 = complete hindlimb paralysis and forelimb paresis/paralysis, as previously described [24, 27]. We also evaluated additional neurological parameters with “yes” or “no” answers including the lack of grooming, eye and/or nose discharge, lethargic behavior, tail paresis and orbital tightening. Due to the risk of viral transmission to other mice, control mice and MHV infected mice were housed in separate cages and separate racks. Research staff were responsible for changing MHV infected mice cages bedding, water, and food weekly and were trained by the CU-AMC vivarium on how to do so. Mice were euthanized according to the experimental timeline, with eight mice euthanized for spinal cord and bladder collection one week post infection, and four mice euthanized at 4 weeks post infection. Humane endpoints were outlined ahead of time for this study. Mice showing weight loss or signs of distress were supplemented with food and non-wetting hydration HydroGel packets (ClearH_2_O, Westbrook, ME) in the bottom of the cage. NSAIDs could not be used in these animals to relieve symptoms/discomfort as they would have stopped the progression of the disease necessary to study this condition. Any mice that exhibited the following signs of pain and distress were to be euthanized: quadriparesis/paralysis or prolonged impaired ambulation (unable to reach food or water), severe encephalitis, signs of dehydration, prolonged lack of appetite, bleeding from any orifice, or self-induced trauma. For euthanasia, mice were deeply anesthetized with sodium pentobarbital followed by decapitation. These procedures are consistent with recommendations of the Panel on Euthanasia of the American Veterinary Medical Association [133]. None of the mice in this study were found dead or met the criteria for humane endpoints. All mice were euthanized at the designated experimental time points.

### Tissue isolation and total RNA extraction

The lumbosacral (L6-S2) spinal cord (SC) segments and urinary bladders (UB) were collected during acute stage of infection (1 wk), and at the first peak of demyelination (4 wks, N = 4 per group per time point). Isolated tissues were immediately placed on dry ice for freezing, and then stored at -80 C. Total RNA was isolated using the Qiagen RNeasy MINI kit (Qiagen, Clifton Hill, Victoria, Australia) according to the manufacturer’s instructions. The amount of total RNA was quantified on the NanoDrop Microvolume Spectrophotometer (ThermoFisher Scientific, Walttham, MA), and the RNA quality was assessed on an RNA denaturing gel following previously established protocol [134].

### nCounter neuroinflammation panel experiment

nCounter Neuroinflammation panel (NanoString Technologies Inc., Seattle, WA) is based on the nCounter barcoding technology that allows for direct quantification of 770 neuroinflammation-related transcripts using an automated platform. It simultaneously measures the relative abundance of 5 CNS cell types and 14 peripheral immune cell types by using the unique cell profiling features, and provides a comprehensive assessment of 23 neuro-inflammatory pathways and processes. We chose this panel for our specimen analysis to identify the cell types, pathways and gene expression profiles affected by coronavirus-induced inflammation in the CNS, and compare them to the UB genes during neurogenic LUTS development. Total RNA isolated from each specimen was diluted to 10 ng/μl, and 5 μl per sample was loaded on the plate. RNA was hybridized with gene-specific reporter and capture probes (controls preloaded in the neuroinflammation plate) at 65°C for 18 h, and then processed on the nCounter Prep station. The final readings were acquired using nCounter Sprint scanner (NanoString Technologies Inc., Seattle, WA).

### Statistical analyses

Statistical analyses of raw datasets were completed using the ROSALIND online platform from OnRamp (https://rosalind.onramp.bio/, San Diego, CA). Read distribution percentages, identity heat maps and sample multi-dimensional scaling (MDS) plots were generated as part of the quality control (QC) step. Samples used for analysis had the following QC metrics: % FOV captured: 1.0±0.009, binding density: 0.631±0.0.59, positive control linearity: 119±4.954. Normalization was completed by dividing counts within a lane by the geometric mean of the normalizer probes from the same lane. We used nCounter Advanced Analysis 2.0 Plugin for nSolver Software available at Nano AA User Manual.pdf (hubspotusercontent40.net) to perform statistical analyses of the complex datasets obtained from the transcriptomic assay. The assay had 8 negative controls and 6 positive controls included in the Neuroinflammatory panel. There were also 8 housekeeping genes included in the assay which were used for normalization of raw data (*Supt71; Cnot10; Tbp; Aars; Tada2b; Lars; Xpnpep1; Csnk2a2*). The assay included a total of 13 housekeeping genes, but the program chose the ones that had the highest/most equal expression between all the samples. The following parameters were analyzed in this study: MDS, gene set analysis (GSA) including the global significance score (GSS) and directed significance score (DSS), cell type scores, and normalized log2 expression. Multi-dimensional scaling is a statistical technique that creates a map displaying a relative position of several genes (clustering) between the groups. The GSA is used to summarize the combined t-test of differential expression of all the genes in a selected pathway for the covariate of interest, as defined by databases including WikiPathways, REACTOME, MSigDB, and Gene Ontology. It evaluates whether gene expression in a pathway is changing in comparison to the baseline. The GSS shows the overall changes, regardless of the direction, while the DSS displays the changes in the specific pathway as an up- or down-regulation in expression level. The cell type scores calculate the abundance of specific cell type populations using marker genes that are stably expressed in given cell types. ROSALIND automatically filters the data to include only results with a p value ≤0.05. Normalization of gene expression was also done automatically by ROSALIND (as described on p. 41 of the nCounter® Advanced Analysis 2.0 User Manual). Differential expression was calculated from the normalized data. Fold changes and p values were calculated using the fast method (nCounter® Advanced Analysis 2.0 User Manual, p. 50). The Benjamin-Hochberg method of estimating false discovery rates was used for p value adjustment.

After preliminary analyses of the raw datasets, and due to a large number of the significantly changed genes, SC genes were filtered by a +/-fold change of 10, and a p value ≤0.05; while the UB genes were filtered by a +/-fold change of 1.5, and p≤0.05. Fold changes and p values were calculated using criteria provided by NanoString Technologies Inc. [23]. Differences between the groups were considered statistically significant at *p*≤0.05.

The MDS plot, cell type profiling heat maps, and the sample correlation heat maps were generated in the ROSALIND platform based on the selected datasets. For the cell type profiling heat map, the colors represent Z-score, with values that fall below the mean of the internal control population being bluer, and the values that fall above the mean being more orange. In the sample correlation heatmap, a darker blue indicates a closer correlation. The figures showing the mouse CSS scores, average weights of the mice and GSS were created using Microsoft Excel (Version 2201. Redmond, WA). Box plots of cell type scores, and box plots of normalized log2 gene expression were created in IBM SPSS (IBM Statistics for Windows. Version 28.0.1.0, Armonk, NY: IBM Corp.) using raw data values acquired through ROSALIND.

## Supporting information

S1 Table Expression of the genes in spinal cord specimens in CIE 1 wk group based on signaling pathways. (DOCX)

S2 Table Expression of the genes in spinal cord specimens from CIE 4 wks mice grouped by signaling pathways. (DOCX)

S3 Table Expression of the genes in the urinary bladder of mice from CIE 1 wk group based on signaling pathways. (DOCX)

S4 Table Expression of the genes in urinary bladder specimens of CIE mice at 4 wks after infection grouped by signaling pathways. (DOCX)
