# Supplementary material for: Differential transcriptomic changes in the central nervous system and urinary bladders of mice infected with a coronavirus
Source: PLoS One. 2022 Dec 9;17(12):e0278918. doi: 10.1371/journal.pone.0278918 (PMC9733897; doi:10.1371/journal.pone.0278918)
Supplement: S1 Table — (DOCX) [file pone.0278918.s001.docx]

| **Pathway** | **Global Significance Score** | **Directed Significance Score** | **Gene** | **Fold Change** |
| --- | --- | --- | --- | --- |
| **Astrocyte Function** | 12.1759 | 11.9318 | *Sb100* | 821.502 |
|  |  |  | *Cxcl9* | 789.535 |
|  |  |  | *Cxcl10* | 334.103 |
|  |  |  | *Slc6a1* | 213.75 |
|  |  |  | *Tgfa* | 134.5 |
|  |  |  | *Gbp2* | 125.131 |
|  |  |  | *B3gnt5* | 103.5 |
|  |  |  | *Serpina3n* | 82.3976 |
|  |  |  | *Slfn8* | 69.675 |
|  |  |  | *C4a* | 43.2953 |
|  |  |  | *Ccl2* | 41.6115 |
|  |  |  | *Slc1a3* | 38.82887 |
|  |  |  | *Psmb8* | 36.2307 |
|  |  |  | *H2-t23* | 32.4447 |
|  |  |  | *Kcnj10* | 25.681 |
|  |  |  | *Smigo2* | 22.3751 |
|  |  |  | *Cp* | 20.9413 |
|  |  |  | *Timp1* | 18.9456 |
|  |  |  | *Sphk1* | 15.5358 |
|  |  |  | *Gpoe* | 13.1201 |
|  |  |  | *Padi2* | -10.6464 |
|  |  |  | *S1pr2* | 10.3682 |
|  |  |  | *Srgn* | 9.68358 |
| **Inflammatory Signaling** | 11.5885 | 11.4049 | *Ccl5* | 1741.48 |
|  |  |  | *S100b* | 821.502 |
|  |  |  | *Cxcl10* | 334.103 |
|  |  |  | *Gpb2* | 125.131 |
|  |  |  | *Cyp7b1* | 123.5 |
|  |  |  | *Ccl7* | 91.3929 |
|  |  |  | *Trpa1* | 75.4998 |
|  |  |  | *Cd72* | 71.6786 |
|  |  |  | *Ch25h* | 54.4998 |
|  |  |  | *Ccl2* | 41.6115 |
|  |  |  | *Ltb* | 36.3751 |
|  |  |  | *Irf7* | 36.2983 |
|  |  |  | *Psmb8* | 36.2307 |
|  |  |  | *Mpeg* | 35.5981 |
|  |  |  | *H2-t23* | 32.4447 |
|  |  |  | *Ptpn6* | 25.9219 |
|  |  |  | *Stat1* | 24.8353 |
|  |  |  | *Btk* | 24.675 |
|  |  |  | *Fcgr1* | 21.3246 |
|  |  |  | *Irf1* | 20.423 |
|  |  |  | *Tmem173* | 16.9583 |
|  |  |  | *Il2rg* | 16.5853 |
|  |  |  | *Fcer1g* | 16.0239 |
|  |  |  | *Tnfsf13b* | 15.0938 |
|  |  |  | *Fcgre3* | 14.8542 |
|  |  |  | *Msr1* | 13.912 |
|  |  |  | *Oas1g* | 12.671 |
|  |  |  | *Cd74* | 12.3077 |
|  |  |  | *Ddx58* | 11.2704 |
| **Lipid Metabolism** | 10.8986 | 10.4016 | *Fah2* | 324.249 |
|  |  |  | *Gal3st1* | 110.0 |
|  |  |  | *Ugt8a* | 47.4232 |
|  |  |  | *Lsr* | 27.1071 |
|  |  |  | *Apoe* | 13.1201 |
|  |  |  | *Pla2g4* | 10.5833 |
| **Insulin Signaling** | 10.4003 | 9.9006 | *Nefl* | 675.251 |
|  |  |  | *Psmb8* | 36.2307 |
|  |  |  | *Kit* | 33.9999 |
|  |  |  | *Il2rg* | 16.5853 |
| **Angiogenesis** | 10.1154 | 7.6751 | *Nefl* | 675.251 |
|  |  |  | *Psmb8* | 36.2307 |
|  |  |  | *Kit* | 33.9999 |
|  |  |  | *Vav1* | 30.1294 |
|  |  |  | *Il2rg* | 16.5853 |
|  |  |  | *Sphk1* | 15.5358 |
| **Oligodendrocyte Function** | 10.0987 | 10.0721 | *Gjb1* | 487.251 |
|  |  |  | *Bcas1* | 380.85 |
|  |  |  | *Fa2h* | 324.249 |
|  |  |  | *Plp1* | 278.0 |
|  |  |  | *Pllp* | 254.249 |
|  |  |  | *Mobp* | 189.499 |
|  |  |  | *Mal* | 159.195 |
|  |  |  | *Mag* | 116.7 |
|  |  |  | *Gal3st1* | 110.0 |
|  |  |  | *Ugt8a* | 47.4232 |
|  |  |  | *Myrf* | 46.1251 |
|  |  |  | *Sox10* | 30.4772 |
|  |  |  | *PLXNB3* | 30.2045 |
|  |  |  | *CNP* | 46.1251 |
|  |  |  | *LINGO1* | 26.2501 |
|  |  |  | *ENPP6* | 17.2499 |
|  |  |  | *S1PR5* | 11.5 |
|  |  |  | *Pla2g4a* | 10.5833 |
| **Cytokine Signaling** | 9.6868 | 8.8065 | *Ccl5* | 821.502 |
|  |  |  | *Cxcl9* | 789.535 |
|  |  |  | *Nefl* | 675.251 |
|  |  |  | *Ngfr* | 343.999 |
|  |  |  | *Cxcl10* | 334.103 |
|  |  |  | *Ccr5* | 324.499 |
|  |  |  | *Ccl7* | 91.3929 |
|  |  |  | *Il21r* | 84.2503 |
|  |  |  | *Il1rn* | 63.2143 |
|  |  |  | *Ccl2* | 41.6115 |
|  |  |  | *Il1rap* | 41.1249 |
|  |  |  | *Ccl4* | 41.0625 |
|  |  |  | *Cx3cr1* | 38.5963 |
|  |  |  | *Ltb* | 36.3751 |
|  |  |  | *Psmb8* | 36.2307 |
|  |  |  | *Kit* | 33.9999 |
|  |  |  | *Vav1* | 30.1294 |
|  |  |  | *Casp1* | 26.4063 |
|  |  |  | *Ptpn6* | 25.9219 |
|  |  |  | *Stat1* | 24.8353 |
|  |  |  | *Cdkn1a* | 17.4455 |
|  |  |  | *Il2rg* | 16.5853 |
|  |  |  | *Tnfsf13b* | 15.0938 |
|  |  |  | *Ccl3* | 10.4063 |
| **Adaptive Immune Response** | 9.6868 | 9.1004 | *Nefl* | 675.251 |
|  |  |  | *Tubb3* | 484.583 |
|  |  |  | *Cd3d* | 251.499 |
|  |  |  | *Cd3g* | 190.874 |
|  |  |  | *Klrd1* | 139.75 |
|  |  |  | *Cd8a* | 102.75 |
|  |  |  | *Cd72* | 71.6786 |
|  |  |  | *Siglec1* | 70.7501 |
|  |  |  | *Lair1* | 42.8624 |
|  |  |  | *Cd3e* | 39.6429 |
|  |  |  | *Psmb8* | 36.2307 |
|  |  |  | *Kit* | 33.9999 |
|  |  |  | *Tubb4a* | 32.4643 |
|  |  |  | *H2-t23* | 32.4447 |
|  |  |  | *Mapk10* | 31.6271 |
|  |  |  | *Was* | 30.3215 |
|  |  |  | *Kif2c* | 30.2499 |
|  |  |  | *Vav1* | 30.1294 |
|  |  |  | *Ctss* | 29.7331 |
|  |  |  | *Ptprc* | 28.6356 |
|  |  |  | *Ptpn6* | 25.9219 |
|  |  |  | *Btk* | 24.675 |
|  |  |  | *Fcgr1* | 21.3246 |
|  |  |  | *Pik3cg* | 18.0001 |
|  |  |  | *Cdkn1a* | 17.4455 |
|  |  |  | *Il2rg* | 16.5853 |
|  |  |  | *Fcer1g* | 16.0239 |
|  |  |  | *Sell* | 15.4059 |
|  |  |  | *Fcgr3* | 14.8542 |
|  |  |  | *Cd69* | 13.9583 |
|  |  |  | *Nfkbie* | 13.2589 |
|  |  |  | *Klrk1* | 12.8026 |
|  |  |  | *Cd74* | 12.3077 |
|  |  |  | *Cd8b1* | 12.225 |
|  |  |  | *Pik3r5* | 10.65 |
| **Matrix Remodeling** | 9.5624 | 9.3803 | *Mag* | 116.7 |
|  |  |  | *Cd8a* | 102.75 |
|  |  |  | *Nlgn1* | 89.25 |
|  |  |  | *Cd6* | 87.2498 |
|  |  |  | *Siglec1* | 70.7501 |
|  |  |  | *Reln* | 60.6136 |
|  |  |  | *Spp1* | 481362 |
|  |  |  | *Itgax* | 40.5 |
|  |  |  | *H2-t23* | 32.4447 |
|  |  |  | *Ctss* | 29.7331 |
|  |  |  | *Ptprc* | 28.6356 |
|  |  |  | *Timp1* | 18.9456 |
|  |  |  | *Sell* | 15.4059 |
|  |  |  | *Cd8b1* | 122.225 |
| **Microglia Function** | 9.5277 | 8.2067 | *Ccl5* | 1741.48 |
|  |  |  | *Zbp1* | 942.253 |
|  |  |  | *Pacsin1* | 368.25 |
|  |  |  | *Cxcl10* | 334.103 |
|  |  |  | *Ccr5* | 324.499 |
|  |  |  | *Slamf8* | 162.0 |
|  |  |  | *Tgfa* | 134.5 |
|  |  |  | *Kcnd1* | 128.5 |
|  |  |  | *Chn2* | 87.4999 |
|  |  |  | *Pmp22* | 69.0807 |
|  |  |  | *Il1rn* | 63.2143 |
|  |  |  | *Stmn1* | 56.9617 |
|  |  |  | *Spint1* | 52.75 |
|  |  |  | *Lst1* | 48.4999 |
|  |  |  | *Spp1* | 48.1362 |
|  |  |  | *Lair1* | 42.8624 |
|  |  |  | *Abcc3* | 42.5 |
|  |  |  | *Ccl4* | 41.0625 |
|  |  |  | *Tlr2* | 40.7501 |
|  |  |  | *Itgax* | 40.5 |
|  |  |  | *Cx3cr1* | 38.5963 |
|  |  |  | *Ppffia4* | 38.0001 |
|  |  |  | *Psmb8* | 36.2307 |
|  |  |  | *Slamff9* | 31.9444 |
|  |  |  | *Ctss* | 29.7331 |
|  |  |  | *Snca* | 25.3707 |
|  |  |  | *Tlr7* | 24.993 |
|  |  |  | *Stat1* | 24.8353 |
|  |  |  | *Rtn4rl1* | 23.7272 |
|  |  |  | *Ncaph* | 22.875 |
|  |  |  | *Cytip* | 20.2688 |
|  |  |  | *Rrm2* | 19.0313 |
|  |  |  | *Nlrp3* | 18.375 |
|  |  |  | *Tmem173* | 16.9583 |
|  |  |  | *Mef2c* | -16.532 |
|  |  |  | *Rab6b* | 16.2041 |
|  |  |  | *Tspan18* | 16.1207 |
|  |  |  | *Ak1* | -15.8661 |
|  |  |  | *Slc2a1* | 15.6169 |
|  |  |  | *Lrrc3* | 15.25 |
|  |  |  | *P2rx7* | 14.2627 |
|  |  |  | *Mertk* | 13.6498 |
|  |  |  | *Tmem144* | 13.6257 |
|  |  |  | *Apoe* | 13.1201 |
|  |  |  | *Hcar2* | 13.05 |
|  |  |  | *Bcl2* | 13.0236 |
|  |  |  | *Oas1g* | 12.671 |
|  |  |  | *Lfng* | 12.4286 |
|  |  |  | *Fscn1* | 10.6153 |
|  |  |  | *Ccl3* | 10.4063 |
|  |  |  | *Kcnk13* | 10.35 |
|  |  |  | *Ldha* | -10.0162 |
