# Supplementary material for: Differential transcriptomic changes in the central nervous system and urinary bladders of mice infected with a coronavirus
Source: PLoS One. 2022 Dec 9;17(12):e0278918. doi: 10.1371/journal.pone.0278918 (PMC9733897; doi:10.1371/journal.pone.0278918)
Supplement: S2 Table — (DOCX) [file pone.0278918.s002.docx]

| **Pathway** | **Global Significance Score** | **Directed Significance Score** | **Gene** | **Fold Change** |
| --- | --- | --- | --- | --- |
| **Lipid Metabolism** | 12.3767 | 12.4304 | *Fa2h* | 290.0 |
|  |  |  | *Gal3st1* | 166.0 |
|  |  |  | *Ugt8a* | 75.3079 |
|  |  |  | *Apoe* | 37.3403 |
|  |  |  | *Gdpd2* | 17.2499 |
|  |  |  | *Lsr* | 15.8571 |
|  |  |  | *Hpgds* | 10.9032 |
| **Oligodendrocyte** | 11.2682 | 11.0475 | *Plp1* | 1930.56 |
|  |  |  | *Mog* | 1414.03 |
|  |  |  | *Mobp* | 1242.22 |
|  |  |  | *Bcas1* | 1195.42 |
|  |  |  | *Opalin* | 644.028 |
|  |  |  | *Gjb1* | 597.999 |
|  |  |  | *Mag* | 483.399 |
|  |  |  | *Myrf* | 346.5 |
|  |  |  | *Pllp* | 334.333 |
|  |  |  | *Fa2h* | 290.0 |
|  |  |  | *Enpp6* | 209.666 |
|  |  |  | *Gal3st1* | 166.0 |
|  |  |  | *Mal* | 156.46 |
|  |  |  | *S1pr5* | 115.667 |
|  |  |  | *Utg8a* | 75.3079 |
|  |  |  | *Lingo1* | 55.0 |
|  |  |  | *Cnp* | 52.418 |
|  |  |  | *Gpr62* | 48.1112 |
|  |  |  | *Plxnb3* | 43.9092 |
|  |  |  | *Sox10* | 27.606 |
| **Neurons & Neurotransmission** | 9.776 | 9.4235 | *Nefl* | 1313.04 |
|  |  |  | *Slc6a1* | 988.666 |
|  |  |  | *Gria4* | 810.332 |
|  |  |  | *Nptx1* | 791.667 |
|  |  |  | *Gjb1* | 597.999 |
|  |  |  | *Slc17a6* | 408.001 |
|  |  |  | *Tubb3* | 342.222 |
|  |  |  | *Nlgn1* | 323.0 |
|  |  |  | *Syn2* | 288.5 |
|  |  |  | *Gria2* | 273.667 |
|  |  |  | *Grm3* | 234.782 |
|  |  |  | *Tgfa* | 228.999 |
|  |  |  | *Kcnj10* | 195.31 |
|  |  |  | *Syp* | 153.108 |
|  |  |  | *Tubb4a* | 133.822 |
|  |  |  | *S1pr5* | 115.667 |
|  |  |  | *Ppfia4* | 104.0 |
|  |  |  | *Camk4* | 75.3333 |
|  |  |  | *Slc1a3* | 61.0856 |
|  |  |  | *Arc* | 57.0 |
|  |  |  | *Kcnd1* | 53.0 |
|  |  |  | *Gria1* | 48.842 |
|  |  |  | *Reln* | 43.3635 |
|  |  |  | *Rbfox3* | 38.7367 |
|  |  |  | *Apoe* | 37.3403 |
|  |  |  | *Bdnf* | 36.3333 |
|  |  |  | *Grin2a* | 35.3334 |
|  |  |  | *Kcnk13* | 25.6 |
|  |  |  | *Dlg4* | 23.9445 |
|  |  |  | *Nlgn2* | 16.952 |
|  |  |  | *Gzma* | 15.0 |
|  |  |  | *Pllekhb1* | 12.1978 |
|  |  |  | *Gja1* | 11.3351 |
| **Astrocyte Function** | 9.0757 | 8.6947 | *S100b* | 1712.28 |
|  |  |  | *Slc6a1* | 988.666 |
|  |  |  | *Grm3* | 234.782 |
|  |  |  | *Tgfa* | 228.999 |
|  |  |  | *Kcnj10* | 195.31 |
|  |  |  | *Slc1a3* | 61.0856 |
|  |  |  | *Cxcl9* | 56.2858 |
|  |  |  | *Serpina3n* | 46.0909 |
|  |  |  | *Apoe* | 37.3403 |
|  |  |  | *B3gnt5* | 32.3333 |
|  |  |  | *Cxcl10* | 24.4117 |
|  |  |  | *C4a* | 22.993 |
|  |  |  | *Slfn8* | 19.3999 |
|  |  |  | *Nwd1* | 19.1601 |
|  |  |  | *Gdpd2* | 17.2499 |
|  |  |  | *Amigo2* | 12.6667 |
|  |  |  | *Sox9* | 12.1846 |
|  |  |  | *Gja1* | 11.3351 |
| **Insulin Signaling** | 9.0291 | 8.6865 | *Nefl* | 1313.04 |
|  |  |  | *Grin2a* | 35.3334 |
|  |  |  | *Dlg4* | 23.9445 |
|  |  |  | *Kit* | 21.6667 |
|  |  |  | *Atp6v1a* | 11.2296 |
| **Angiogenesis** | 8.563 | 7.4865 | *Nefl* | 1313.04 |
|  |  |  | *Grin2a* | 35.3334 |
|  |  |  | *Dlg4* | 23.9445 |
|  |  |  | *Kit* | 21.6667 |
|  |  |  | *Vav1* | 12.8276 |
| **Microglia Function** | 8.2389 | 7.3802 | *Pacsin1* | 943.998 |
|  |  |  | *Ccl5* | 313.667 |
|  |  |  | *Tgfa* | 228.999 |
|  |  |  | *Chn2* | 171.0 |
|  |  |  | *Cx3cr1* | 115.231 |
|  |  |  | *Ppfia* | 104.0 |
|  |  |  | *Tmem144* | 70.5977 |
|  |  |  | *Zbp1* | 69.3335 |
|  |  |  | *Stmn1* | 66.6573 |
|  |  |  | *Kcnd1* | 53.0 |
|  |  |  | *Tlr2* | 44.6668 |
|  |  |  | *Slamf8* | 42.0001 |
|  |  |  | *Cst7* | 40.4829 |
|  |  |  | *Ccr5* | 40.0001 |
|  |  |  | *Apoe* | 37.3503 |
|  |  |  | *Ctss* | 36.365 |
|  |  |  | *Spp1* | 34.2989 |
|  |  |  | *Itgax* | 33.9999 |
|  |  |  | *Rab6b* | 30.8911 |
|  |  |  | *Gpr34* | 30.5386 |
|  |  |  | *Cd83* | 30.434 |
|  |  |  | *Lair1* | 27.2007 |
|  |  |  | *Trem2* | 26.7675 |
|  |  |  | *Snca* | 26.2068 |
|  |  |  | *Kcnk13* | 25.6 |
|  |  |  | *Cxcl10* | 24.4117 |
|  |  |  | *Spint1* | 23.6666 |
|  |  |  | *Hcar2* | 20.6 |
|  |  |  | *Abcc3* | 17.0 |
|  |  |  | *Pmp22* | 16.6815 |
|  |  |  | *Lst1* | 16.3334 |
|  |  |  | *Lrrc3* | 15.7778 |
|  |  |  | *Slc2a1* | 13.8387 |
|  |  |  | *Tlr7* | 13.5086 |
|  |  |  | *Tspan18* | 12.3042 |
|  |  |  | *Fabp5* | 11.8121 |
|  |  |  | *Atp6v1a* | 11.2296 |
|  |  |  | *Rtn4rl1* | 11.1818 |
|  |  |  | *Clstn1* | 10.9891 |
|  |  |  | *Gna15* | 10.7692 |
|  |  |  | *Cnaph* | 10.5 |
|  |  |  | *P2ry12* | 10.437 |
|  |  |  | *Fscn1* | 10.276 |
|  |  |  | *Slamf9* | 10.037 |
| **Matrix Remodeling** | 7.7586 | 6.9357 | *Mag* | 483.399 |
|  |  |  | *Nlgn1* | 323.0 |
|  |  |  | *Reln* | 43.3635 |
|  |  |  | *Ctss* | 36.365 |
|  |  |  | *Spp1* | 34.2989 |
|  |  |  | *Itgax* | 33.9999 |
|  |  |  | *Nlgn2* | 16.952 |
|  |  |  | *Cd8a* | 14.6667 |
|  |  |  | *Siglec1* | 12.3333 |
| **Adaptive Immune Response** | 7.7586 | 7.2366 | *Nefl1* | 1313.04 |
|  |  |  | *Tubb3* | 342.222 |
|  |  |  | *Tubb4a* | 133.822 |
|  |  |  | *Mapk10* | 71.2713 |
|  |  |  | *Cd3g* | 45.2501 |
|  |  |  | *3d3d* | 39.6668 |
|  |  |  | *Ctss* | 36.365 |
|  |  |  | *Grin2a* | 35.3334 |
|  |  |  | *Lair1* | 27.2007 |
|  |  |  | *Trem2* | 26.7675 |
|  |  |  | *Dlg4* | 239445 |
|  |  |  | *Cd72* | 21.9286 |
|  |  |  | *Kit* | 21.6667 |
|  |  |  | *Btk* | 18.0001 |
|  |  |  | *Klrd1* | 17.6667 |
|  |  |  | *Cd8a* | 14.6667 |
|  |  |  | *Ptpn6* | 14.0 |
|  |  |  | *Vav1* | 12.8276 |
|  |  |  | *Fcgr3* | 12.6111 |
|  |  |  | *Siglec1* | 12.3333 |
|  |  |  | *Pik3cg* | 11.6667 |
|  |  |  | *Cd74* | 10.3298 |
|  |  |  | *Was* | 10.2857 |
| **Autophagy** | 7.3624 | 6.5921 | *Clec7a* | 84.3333 |
|  |  |  | *Mapk10* | 71.2713 |
|  |  |  | *Vav1* | 12.8276 |
|  |  |  | *Fcgr3* | 12.6111 |
|  |  |  | *Siglec1* | 12.3333 |
|  |  |  | *Was* | 10.2857 |
