# Supplementary material for: Differential transcriptomic changes in the central nervous system and urinary bladders of mice infected with a coronavirus
Source: PLoS One. 2022 Dec 9;17(12):e0278918. doi: 10.1371/journal.pone.0278918 (PMC9733897; doi:10.1371/journal.pone.0278918)
Supplement: S3 Table — (DOCX) [file pone.0278918.s003.docx]

| **Pathway** | **Global Significance Score** | **Directed Significance Score** | **Gene** | **Fold Change** |
| --- | --- | --- | --- | --- |
| **Matrix remodeling** | 3.2838 | -1.9109 | *Col6a3* | -2.70572 |
|  |  |  | *Siglec1* | 2.55714 |
|  |  |  | *Itga7* | -2.2222 |
|  |  |  | *Ttr* | -1.5901 |
|  |  |  | *Fbln5* | -1.91115 |
|  |  |  | *Ctss* | 1.67138 |
|  |  |  | *Pdpn* | -1.5901 |
|  |  |  | *1cam2* | -1.57353 |
|  |  |  | *Cd86* | 1.50555 |
| **Astrocyte Function** | 2.7958 | -1.472 | *Fkbp5* | 4.25253 |
|  |  |  | *Itga7* | -2.2222 |
|  |  |  | *Fbln5* | 1.91115 |
|  |  |  | *Hspb1* | -1.74996 |
|  |  |  | *Gbp2* | -1.68899 |
|  |  |  | *C4a* | 1.55506 |
| **Microglia Function** | 2.6951 | -0.9185 | *Ccl5* | 2.92106 |
|  |  |  | *Igf1* | -2.82179 |
|  |  |  | *Zbp1* | 2.76596 |
|  |  |  | *Col6a3* | -2.70572 |
|  |  |  | *Il1rn* | 2.07247 |
|  |  |  | *Serpinf1* | -1.9863 |
|  |  |  | *Maff* | -1.92652 |
|  |  |  | *Mertk* | 1.9027 |
|  |  |  | *Nrp2* | -1.87244 |
|  |  |  | *Npnt* | -1.86066 |
|  |  |  | *Asb2* | -1.81658 |
|  |  |  | *Tmem100* | -1.72881 |
|  |  |  | *Il6ra* | 1.71983 |
|  |  |  | *Ctss* | 1.67138 |
|  |  |  | *Tgm2* | -1.62924 |
|  |  |  | *Crip1* | 1.61614 |
|  |  |  | *Ctse* | 1.60225 |
|  |  |  | *Tmem204* | -1.58173 |
|  |  |  | *Eef2k* | 1.5579 |
|  |  |  | *Tspan18* | -1.5431 |
|  |  |  | *Cd86* | 1.50555 |
| **Neurons & Neurotransmission** | 2.5895 | 1.283 | *Gzma* | 5.03032 |
|  |  |  | *Cd163* | 3.47798 |
|  |  |  | *Calr* | -1.56948 |
| **Wnt** | 2.4152 | -2.238 | *Serpinf1* | -1.9863 |
| **Autophagy** | 2.4081 | -1.2083 | *Siglec1* | 2.55714 |
|  |  |  | *Mertk* | 1.9027 |
|  |  |  | *Cnn2* | -1.8084 |
|  |  |  | *Tgm2* | -1.62924 |
|  |  |  | *Calr* | -1.56948 |
| **Oligodendrocyte Function** | 2.3993 | -1.198 | *Myrf* | -2.33728 |
| **Insulin Signaling** | 2.3441 | 1.1455 | *Igf1* | -2.82179 |
|  |  |  | *Mafb* | 1.56133 |
|  |  |  | *Eef2k* | 1.5579 |
| **Inflammatory Signaling** | 2.2105 | -0.5437 | *Ccl5* | 2.92106 |
|  |  |  | *Mcm5* | -1.78125 |
|  |  |  | *Nfkb2* | -1.7603 |
|  |  |  | *Gbp2* | -1.68899 |
|  |  |  | *Relb* | -1.61917 |
|  |  |  | *Cyp27a1* | 1.5896 |
| **Angiogenesis** | 2.2026 | -1.6214 | *Nrp2* | -1.87244 |
|  |  |  | *Hspb1* | -1.74996 |
