# Supplementary material for: Differential transcriptomic changes in the central nervous system and urinary bladders of mice infected with a coronavirus
Source: PLoS One. 2022 Dec 9;17(12):e0278918. doi: 10.1371/journal.pone.0278918 (PMC9733897; doi:10.1371/journal.pone.0278918)
Supplement: S4 Table — (DOCX) [file pone.0278918.s004.docx]

| **Pathway** | **Global Significance Score** | **Directed Significance Score** | **Gene** | **Fold Change** |
| --- | --- | --- | --- | --- |
| **Matrix Remodeling** | 2.0774 | -0.7714 | *Serpine1* | 3.528 |
|  |  |  | *Cd44* | 2.09091 |
|  |  |  | *Siglec1* | 1.78571 |
|  |  |  | *Ttr* | 1.74074 |
|  |  |  | *Reln* | -1.70349 |
|  |  |  | *Cldn5* | -1.52259 |
| **Insulin Signaling** | 1.8577 | 0.4635 | *Kit* | 1.78571 |
| **Wnt** | 1.7735 | 0.765 | *Jun* | 1.70492 |
| **Notch** | 1.7496 | 1.3452 | *Jun* | 1.7042 |
| **Angiogensis** | 1.7253 | -0.5526 | *Kit* | 1.78571 |
| **NF-kB** | 1.7133 | 0.9682 | *Ptgs2* | 8.918 |
|  |  |  | *Tnfrsf25* | 2.90698 |
|  |  |  | *Traf1* | 1.97059 |
|  |  |  | *Tnfrsf12a* | 1.7508 |
| **Adaptive Immune Response** | 1.6553 | 0.8703 | *Fos* | 2.64312 |
|  |  |  | *Kit* | -1.9246 |
|  |  |  | *Siglec1* | 1.78571 |
|  |  |  | *Jun* | 1.70492 |
|  |  |  | *Assb2* | -1.57851 |
| **Growth Factor** | 1.6377 | -0.2519 | *Fos* | -1.9246 |
|  |  |  | *Siglec1* | 1.78571 |
|  |  |  | *Gadd45g* | 1.7802 |
|  |  |  | *Jun* | 1.70492 |
|  |  |  | *Reln* | -1.70349 |
| **Neurons & Neurotransmission** | 1.6007 | 0.6737 | *Ppfia4* | 2.71712 |
|  |  |  | *Fos* | 2.64312 |
|  |  |  | *Reln* | -1.70349 |
|  |  |  | *Cd163* | 1.69168 |
|  |  |  | *Gria1* | -1.57576 |
| **Autophagy** | 1.5747 | 0.5418 | *Serpine1* | 3.528 |
|  |  |  | *Cd44* | 2.09091 |
|  |  |  | *Siglec1* | 1.78571 |
|  |  |  | *Map1lc3a* | -1.53865 |
